# Long-term proton pump inhibitor usage and the association with pancreatic cancer in Sweden

**DOI:** 10.1007/s00535-019-01652-z

**Published:** 2019-12-06

**Authors:** Nele Brusselaers, Omid Sadr-Azodi, Lars Engstrand

**Affiliations:** 1 Department of Microbiology, Tumor and Cell Biology, Centre for Translational Microbiome Research, Karolinska Institutet, Karolinska Hospital, Visionsgatan 4, 171 64 Stockholm, Sweden; 2 Science for Life Laboratory (SciLifeLab), Stockholm, Sweden; 3 Department of Head and Skin, Faculty of Medicine, Ghent University, Ghent, Belgium; 4 Clinical Epidemiology Division, Department of Medicine Solna, Karolinska Institutet, Stockholm, Sweden; 5 Department of Surgery, St Göran Hospital, Stockholm, Sweden

**Keywords:** Pancreatic neoplasms, Pancreas, 2-Pyridinylmethylsulfinylbenzimidazoles, Gastric acid, PPIs

## Abstract

**Background:**

The long-term safety of proton pump inhibitors (PPIs) is increasingly questioned. The aim of our study was to assess the risk of pancreatic cancer among long-term PPI users in Sweden.

**Methods:**

This population-based nationwide Swedish cohort study including 796,492 adult long-term PPI users has been used to calculate the standardized incidence rate ratios (SIRs) and 95% confidence intervals (CI) for pancreatic cancer, stratifying by indications of use, age, sex, and duration of use. The risk among all 20,210 long-term H2-receptor antagonist users was assessed as comparison.

**Results:**

Pancreatic cancer was found in 1733 long-term PPI users, and 25 H2-receptor antagonist users. For PPI users, the risk of pancreatic cancer was increased overall (SIRs = 2.22; 95% CI 2.12–2.32) and in all subgroup analyses, with the highest risk among PPI-users younger than 40 years (SIR = 8.90, 95% CI 4.26–16.37), and among individuals with a history of *Helicobacter pylori* (SIR = 2.99, 95% CI 2.54–3.49). After the first year after enrolment (during which PPI use may be because of early symptoms of pancreatic cancer), the risk remained increased over time, with SIR = 1.57 (95% CI 1.38–1.76) after 5 years. No associations were found for H2-receptor antagonists (SIR = 1.02, 95% CI 0.66–1.51).

**Conclusions:**

This large study showed an increased risk of pancreatic cancer in long-term users of PPIs in Sweden, in particular among the youngest users.

## Introduction

One of the frequently debated questions in gastro-enterology is if the long-term use of proton pump inhibitors (PPI) is carcinogenic, and if benefits outweigh the risks for all individuals. Proton pump inhibitors are by far the most commonly prescribed medications for almost every problem or discomfort of the upper-gastrointestinal tract, including gastro-esophageal reflux, (prevention of) peptic ulcers, gastroduodenitis, dyspepsia or eradication of *Helicobacter pylori* [1, 2]*.* PPIs are commercialized in the 1980s, and since they are extremely potent in suppressing gastric acid production, close monitoring was initially required with endoscopies and regular follow-up. Nowadays, PPIs are available over-the-counter in many countries, and easily prescribed yet not easily discontinued, leading to a steadily increasing amount of long-term users [1, 3–6]. Noteworthy is that previous studies reported 25–70% of inappropriate use of prescribed PPIs, contributing to polypharmacy and potential drug-drug interactions [1, 7].

Nevertheless, the list of potential side-effects related to long-term PPI use is increasing, including among others, chronic kidney disease, osteoporosis and fractures, *Clostridium difficile* infections, community acquired pneumonia, cardiac diseases, and even increased mortality [8–19]. An increasing number of studies have also investigated the risk of cancer with most evidence existing for gastric, colorectal and pancreatic cancer. The two meta-analyses on gastric cancer (in total including 8 different studies) concluded that there may be an increased risk in particular when used over longer periods of time [20, 21]. Yet, the two meta-analyses evaluating colorectal cancer (including 5 different studies) did not find strong support for an association [22, 23], although 2 more studies have been published since showing a significantly increased risks [24, 25]. For pancreatic cancer, the 12th most common cancer type, with only 8% 5-year survival [26], we have identified 6 case–control studies [27–32] and 1 cohort study [33] of which 3 studies clearly show statistically increased risks (up to 9-times higher than non-users) [27, 29, 30]. Yet, methodological heterogeneity and selection bias may challenge the interpretation of these findings. Therefore, our aim was to assess the risk of pancreatic cancer in our previously used Swedish population-based cohort study [34–36] to compare the risk of pancreatic cancer in including individuals receiving PPI maintenance therapy with the expected risk based on the total Swedish population.

## Methods

This nationwide Swedish population-based cohort study was designed to compare the risk of pancreatic cancer among adults (≥ 18 years) exposed to long-term PPIs compared to the Swedish background population of the same sex, age, and calendar year, following an a-priori established study protocol. The study results are reported according to the STROBE statement (Strengthening the Reporting of Observational Studies in Epidemiology) for cohort studies. This cohort has been described in detail elsewhere [34, 36], and was approved by the Regional Ethical Review Board in Stockholm (2014/1291-31/4). This study has been performed in accordance with the ethical standards laid down in the 1964 Declaration of Helsinki and later amendments, yet informed consent was not required because of the registry-based nature of the data. All individuals, without a history of cancer, were enrolled between 1st July 2005 (start of the Swedish Prescribed Drug Registry) to 31st December 2012, and followed up until the occurrence of any cancer, death or 31st December 2012 (i.e., end of data collection for Cancer Registry), whichever occurred first.

### Exposure

PPI use was defined by the Anatomic Therapeutic Chemical classification (ATC) system code A02BC, as registered in the Swedish Prescribed Drug Registry. Long-term PPI use was defined as ≥ 180 days of exposure to PPI during the study period before onset of any cancer, approximating 1 month per year or more if close to the maximum follow-up of 7.5 years. This total cumulative administered PPI dosage is estimated by adding the defined daily dose per package (DDDp), which takes the potency of the drug into account as well as the prescribed quantity with DDD being the assumed average maintenance dose per day for a drug used for its main indication in adults according to the World Health Organization. For comparison reasons, the risk of pancreatic cancer was also evaluated among all adults who received ≥ 180 days of exposure to H2-receptor antagonists, a drug class with similar indications (ATC code A02BA). All individuals who received both ≥ 180 days of PPIs and ≥ 180 days of H2RA (*N* = 25,726 [36] were excluded from all analyses. PPIs are also available over-the-counter in Sweden since 1999 [6], yet only in small packages (so at a higher price per dose [37], so we can assume that long-term users have the large majority of their PPI doses prescribed.

### Outcome

The outcome was cancer of the pancreas, as (compulsory) registered in the nationwide Cancer Register and defined by the C25 code of the International Classification of Diseases, 10^th^ edition (ICD 10). Pancreatic adenocarcinoma was defined by the histopathological code 096.

### Potential confounders/covariates

Age was grouped by age of first PPI prescription and categorized as 18–40 years, 40–49 years, 50–59 years, 60–69 years, and  ≥ 70 years. Sex was grouped as male or female, and calendar period, as 2005–2006, 2007–2009 and 2010–2012. There was no missing information on these 3 variables.

Potential confounding by indication was evaluated by subgroup analyses by indications for gastric acid suppressive therapy. Although these indications are not known as strong risk factors for pancreatic cancer, a potential protective effect has been described for long-term aspirin or other non-steroidal anti-inflammatory drugs (NSAIDs) [38]. The following 6 subgroups were assessed, as defined earlier based on the Patient Registry and/or Prescribed Drug Registry: [29, 30] (1) gastro-esophageal reflux disease; (2) peptic ulcers; (3) gastroduodenitis; (4) *Helicobacter pylori* eradication/infection, long-term (≥ 180 days during study period) users of (5) aspirin (ATC codes B01AC06, N02BA) or (6) other NSAIDs (ATC code M01A) without any of the selected gastrointestinal indications (including the less prevalent indications dyspepsia, Barrett esophagus and Zollinger-Ellison syndrome, which were recorded in ≤ 5% of the present cohort and therefore not assessed separately). No indication of therapy was registered for approximately 22% of the cohort [34, 36].

An additional subgroup analyses was conducted for individuals with diabetes mellitus, a known risk factor for pancreatic cancer, as defined by the use of any “drugs used in diabetes” (ATC code A10) before the diagnosis of any cancer.

### Statistical analyses

The risk of developing pancreatic cancer was assessed by comparing the observed risk among long-term users of PPIs and the expected risk according to the Swedish background population of the same age, sex and calendar period, by means of standardized incidence rate ratios (SIRs) and 95% confidence intervals (CI) [39]. Expected incidence rates were calculated based on the Swedish Cancer Registry and Total Population Registry [40]. Time of follow-up was calculated from the dispense date of the first prescription of PPI within the study period, until death, cancer or end of study period (December 2012), whichever occurred first. The analyses were stratified by sex, age, indication, and diabetes mellitus as described above, and subgroup analyses were conducted for adenocarcinoma only.

The effect of duration of treatment was assessed by looking at the risk in relation to the time since the first PPI prescription, categorized as < 1.0 year, 1.0–2.9 years, 3.0–4.9 years and more than 5.0 years, as described earlier [35]. These groups are not mutually exclusive (with one individual potential contributing person-time to different groups) as would be the case when using the total estimated accumulated dosage based on the DDD, with the associated risk of immortal time-bias in the groups with higher dosages.

## Results

The characteristics of all 796,492 long-term PPI users are described in Table 1. Approximately 59% of the cohort was female, and 34% was 70 years or older. Long-term aspirin and other NSAIDs use were the most common indications, respectively, in 35% and 30% of the cohort; with relatively more male aspirin users, and more female NSAIDs users. Gastro-esophageal reflux, gastroduodenitis, peptic ulcers, and *Helicobacter pylori* eradication/infection were recorded for, respectively, 25%, 13%, 10%, and 7% of the cohort. Diabetes drugs were used prior cancer diagnosis by 3.5% of women and 5.0% of men.

**Table 1:** Description of the all long-term users of proton pump inhibitors (defined as ≥ 180 days accumulated use) in Sweden during 2005–2012

	Total	Women	Men
	Number (%)	Number (%)	Number (%)
Total	796,492 (100)	465,840 (58.5)	330,652 (41.5)
Age			
< 40 years	88,775 (11.2)	52,780 (11.3)	35,995 (10.9)
40–49 years	103,784 (13.0)	59,440 (12.8)	44,344 (13.4)
50–59 years	155,625 (19.5)	86,756 (18.6)	68,869 (20.8)
60–69 years	177,610 (22.3)	98,012 (21.0)	79,598 (24.1)
≥ 70 years	270,698 (34.0)	168,852 (36.3)	101,846 (30.8)
Calendar period			
2005–2006	437,229 (54.9)	258,700 (55.5)	178,529 (54.0)
2007–2009	227,142 (28.5)	132,730 (28.5)	94,412 (28.6)
2010–2012	132,121 (16.6)	74,410 (16.0)	57,711 (17.5)
Indications of use			
Gastro-esophageal reflux	201,744 (25.3)	109,675 (23.5)	92,069 (27.8)
Peptic ulcers	79,546 (10.0)	40,255 (8.6)	39,291 (11.9)
Gastroduodenitis	104,903 (13.2)	59,654 (12.8)	45,249 (13.7)
*Helicobacter pylori* infection/eradication	58,340 (7.3)	32,082 (6.9)	26,258 (7.9)
Aspirin maintenance therapy	276,941 (34.8)	147,180 (31.6)	129,761 (39.2)
NSAIDs maintenance therapy	241,777 (30.3)	160,632 (34.5)	81,145 (24.5)
Diabetes drugs before cancer diagnosis	33,384 (4.2)	16,531 (3.5)	16,853 (5.0)
Pancreatic cancer	1,733 (0.21)	917 (0.20)	816 (0.25)
Pancreatic adenocarcinoma	1394 (0.18)	742 (0.16)	652 (0.20)

*NSAIDs* non-steroidal anti-inflammatory drugs

### Risk of pancreatic cancer by age and sex

In total, 1733 individuals developed pancreatic cancer, of which 80.4% were adenocarcinomas. The overall SIR of pancreatic cancer among long-term PPI users compared to the Swedish background population, was 2.22 (95% CI 2.12–2.32). The risk estimates were slightly higher for men (SIR = 2.46, 95% CI 2.30–2.64) than women (SIR = 2.04, 95% CI 2.30–2.64) (Table 2). The highest risk estimate was found for the youngest age group (SIR = 8.90, 95% CI 4.26–16.37), with the risk estimates decreasing by age (SIR = 1.81, 95% CI 1.69–1.94 for those ≥ 70 years). The 95% CI of the 4 oldest age-groups did not overlap indicating a statistically significant decrease in risk with increasing age. Similar results were found when restricting to adenocarcinomas.

**Table 2:** The risk of pancreatic cancer and pancreatic adenocarcinoma for all long-term proton pump inhibitor users in Sweden (defined as ≥ 180 days accumulated use), presented as standardized incidence rate ratios (SIRs) and 95% confidence intervals (CI)

	Person years	Total		Adenocarcinoma	
		*N* (%)	SIRs (95% CI)	*N* (%)	SIRs (95% CI)
Total	3,828,553	1733	2.22 (2.12–2.32)	1394	2.26 (2.14–2.38)
Sex					
Women	2,275,213	917	2.04 (1.91–2.17)	742	2.10 (1.95–2.26)
Men	1,553,340	816	2.46 (2.30–2.64)	652	2.46 (2.28–2.66)
Age					
< 40 years	333,857	10	8.90 (4.26–16.37)	7	12.30 (4.93–25.34)
40–49 years	475,113	65	5.93 (4.58–7.56)	54	6.62 (4.97–8.63)
50–59 years	729,786	230	3.36 (2.94–3.82)	187	3.29 (2.84–3.80)
60–69 years	954,060	602	2.46 (2.26–2.66)	520	2.47 (2.27–2.70)
≥ 70 years	1,335,737	826	1.81 (1.69–1.94)	626	1.83 (1.69–1.98)
Indications of use					
Gastro-esophageal reflux	979,793	409	2.06 (1.86–2.27)	331	2.01 (1.87–2.33)
Peptic ulcers	359,158	234	2.59 (2.27–2.94)	182	2.56 (2.20–2.96)
Gastroduodenitis	526,036	307	2.75 (2.45–3.08)	245	2.78 (2.44–3.15)
*Helicobacter pylori* infection/eradication	288,390	160	2.99 (2.54–3.49)	121	2.85 (2.37–3.41)
Long-term aspirin use^a^	497,202	222	1.55 (1.36–1.77)	173	1.56 (1.33–1.81)
Long-term NSAIDs use^a^	571,664	210	2.33 (2.03–2.67)	174	2.40 (2.06–2.79)
Diabetes mellitus drugs	164,936	152	3.76 (3.19–4.41)	118	3.68 (3.05–4.41)
Time since start PPIs					
First year	979,007	689	4.35 (4.03–4.68)	589	4.62 (4.25–5.01)
1–3 years	2,340,030	414	1.28 (1.16–1.40)	331	1.25 (1.12–1.39)
3–5 years	1,159,914	362	1.40 (1.26–1.55)	286	1.40 (1.25–1.58)
> 5 years	747,495	268	1.57 (1.38–1.76)	188	1.45 (1.25–1.67)

*NSAIDs* non-steroidal anti-inflammatory drugs

^a^No other gastrointestinal indications recorded

### Indications of use

**Fig. 1:**
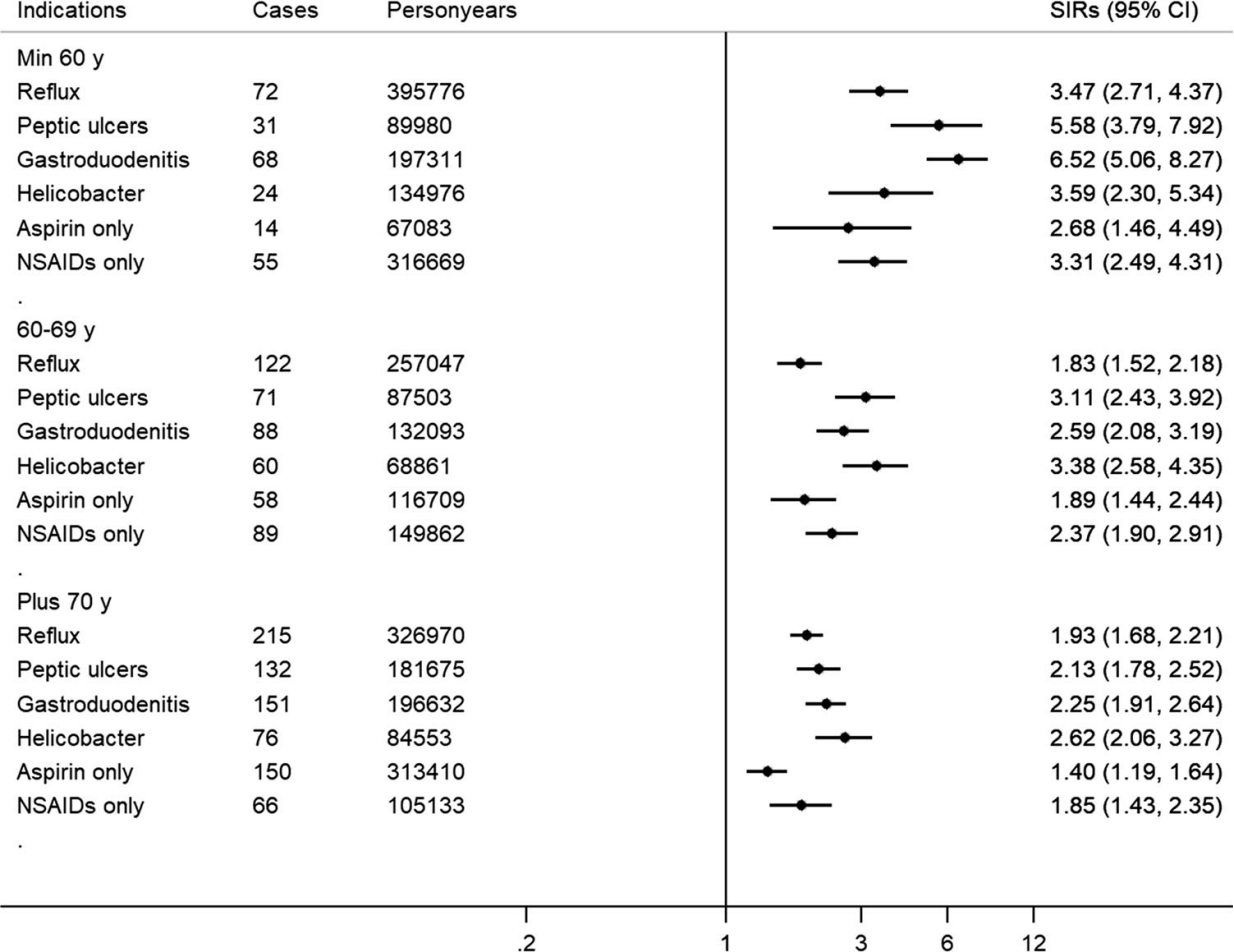
The risk of pancreatic cancer among long-term users of proton pump inhibitors (PPIs) (defined as ≥ 180 days accumulated use) compared to the Swedish background population, for the most common indications of use, stratified per age-group

Compared to the Swedish background population, the risk of pancreatic cancer was higher among PPI long-term users for all indication groups, with the highest SIRs for those with a history of *Helicobacter pylori* infection/eradication (SIR = 2.99, 95% 2.54–3.49), and the lowest risk for long-term aspirin users (SIR = 1.55, 95% 1.36–1.77), again with similar findings for adenocarcinoma only (Table 2). Figure 1 shows the SIRs for each indication per age group, with the youngest 3 age-groups merged to obtain sufficient power. The SIRs were 5–6 times higher for those younger than 60 with peptic ulcers or gastroduodenitis, and the lowest SIR (1.40, 95% 1.19–1.64) was found for long-term aspirin users of 70 years or older.

### Risk among individuals with diabetes mellitus

The SIRs for pancreatic cancer in this group was 3.76 (95% CI 3.19–4.41) and 3.68 (95% CI 3.05–4.41) for pancreatic adenocarcinoma (Table 2).

### Duration of treatment

**Fig. 2:**
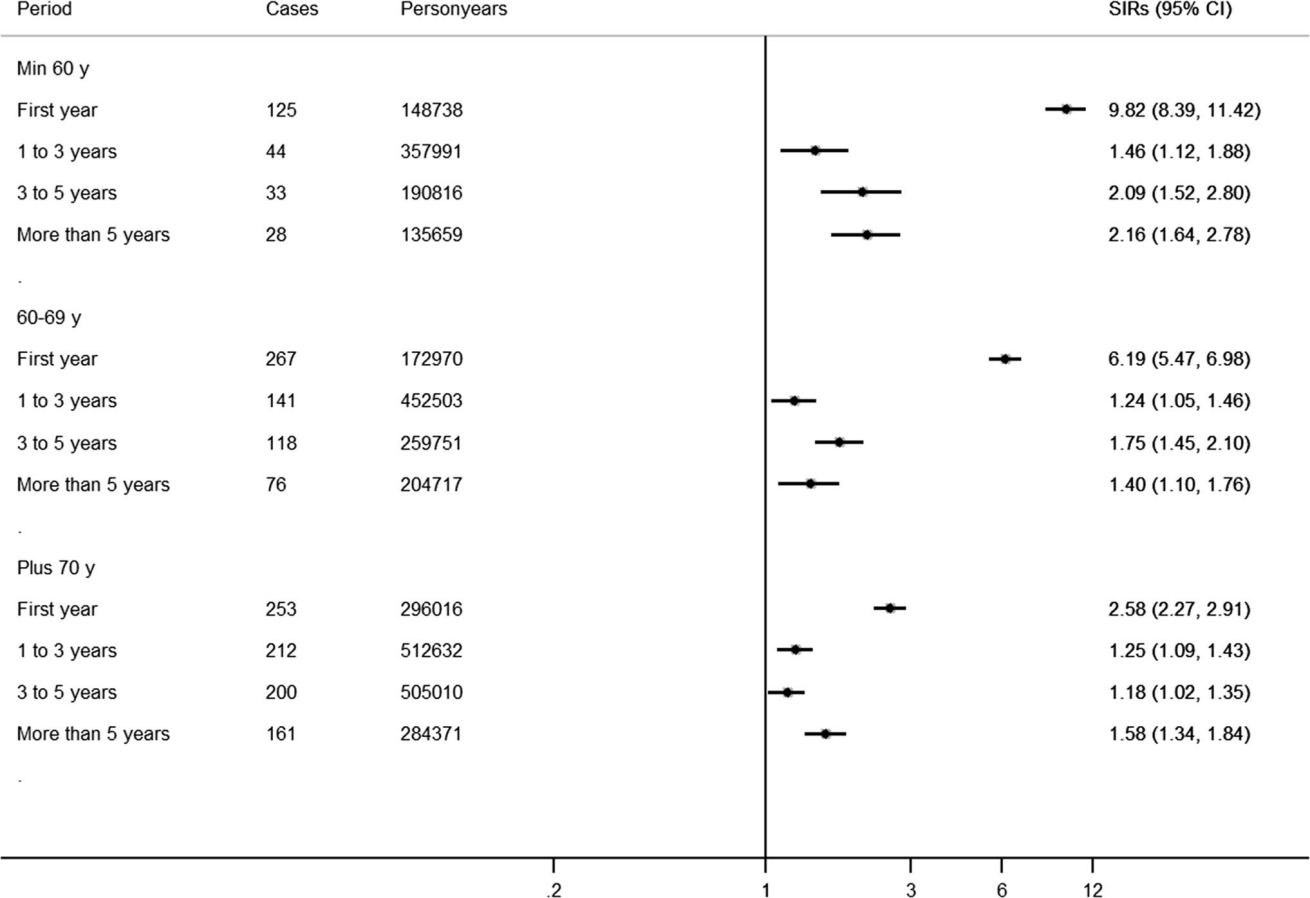
The risk of pancreatic cancer among long-term users of proton pump inhibitors (PPIs) (defined as ≥ 180 days accumulated use) compared to the Swedish background population, stratified per age-group and time since initiation of treatment

During the first year of treatment, the SIRs was 4.35 (95% CI 4.03–4.68), and even 9.82 (95% CI 8.39–11.42) among those younger than 60 years (Fig. 2). After this first year, the risk was still increased, and seemed to increase over time, from SIR = 1.28 (95% CI 1.16–1.40) between 1 and 3 years after treatment initiation to 1.57 (95% CI 1.38–1.76) after 5 years, with again similar findings when restricting to adenocarcinomas. Figure 2 shows the results stratified by age, and this apparent increase over time after the first year was most consistent among those younger than 60 years (from SIR = 1.46, 95% CI 1.12–1.88 to SIR = 2.16, 95% CI 1.64–2.78).

### H2-receptor antagonists

Among the 20,210 long-term users of H2-receptor antagonists, 25 cases of pancreatic cancer occurred for 113,961 person years of follow-up. The analyses did not find evidence for an association, with SIR = 1.02 (95% CI 0.66–1.51) overall, and SIR = 0.92 (0.49–1.57) among women and SIR = 1.17 (0.60–2.04).

## Discussion

This large population-based study assessing long-term use of PPIs showed an overall doubled risk of pancreatic cancer among long-term PPI users compared to Swedish background population, in particular among the younger age-groups. Disregarding the first year of use (which may reflect PPI use because of early symptoms, reverse causality), the risk increased over time since initiating treatment in particular among the youngest age-groups, and the risk increase was shown for all investigated indications of use. There was no association between the H2-receptor antagonist use and pancreatic cancer.

The strengths of this study are the population-based design, the study size with almost 800,000 long-term PPI users with 4 million years of follow-up; and over 20,000 H2-receptor users as comparison group. The, to our knowledge, largest studies previously published describing this association with pancreatic cancer, only included almost 50,000 PPI users from Korea [33]; and 10,500 H2-receptor antagonist users in Australia, Canada and the United States [32]. The maximal duration of follow-up in the previously published cohort study was 12 years, compared to the 7.5 years in the present study. All PPI use in the present study is based on prescriptions, eliminating the risk of misclassification because of recall-bias, in particular because the Swedish Prescribed Drug registry is virtually complete, although no data are available on in-hospital use [41]. Yet, some long-term users may not have been identified if (part of) their PPI use was obtained over-the-counter instead of by prescription [6]. We also lack information on PPI use before July 2005, but previous studies have shown that PPI use overall, and long-term PPI use are increasing over time, and that discontinuation of treatment is often over-looked by physicians [1–5]. Therefore, it is difficult to distinguish between prevalent and incident PPI users, although we assume that many of those exposed in 2005 were already users prior to enrolment in the present study.

We defined long-term use as an accumulated use of 6 months of more, which was considerably stricter than other studies looking at PPI use and the risk of gastric cancer (defined as current use, or minimally 1–2 prescriptions) [20],and pancreatic cancer, all defining PPI use as ≥ 1 prescription [27–33]. Long-term use (> 6 months has only been approved by the Food and Drug Administration for pathological hypersecretory conditions such as the Zollinger–Ellison syndrome; and erosive esophagitis (based on study data < 12 months) [42].

Since long-term PPI use is nevertheless common (approximately 11% of all Swedish adults), using the total Swedish background population to calculate expected risks, may have diluted the overall effect [34, 36, 43]. The total accumulated dosage of PPIs may correlate with an increasing risk of pancreatic cancer. Yet, as described earlier, immortal time-bias is a concern [35], and therefore only data based on time since start of PPI treatment are calculated and presented. The popularity of PPI use also makes it unfeasible to find a comparison group with the same (severity of) symptoms and indications for treatment not receiving treatment with PPIs or H2-receptor antagonists, the most popular yet clearly less common alternative. Although all results are standardized for age, sex and calendar period, residual confounding may remain. Smoking, obesity and diabetes, known risk factors for pancreatic cancer which may also be related to PPI intake, are unfortunately not collected nationwide for calculations of SIRs. According to the Public Health Agency of Sweden, approximately 50% of the Swedish adults is overweight or obese, and 8–11% smoked daily in 2016 [44]. The subgroup analyses on individuals using diabetes medication also showed increased risks of pancreatic cancer, but because of the low proportion of PPI users (4.2%) diabetes cannot explain the increased overall risk. However, it is unlikely that residual confounding would fully explain the increasing risk of pancreatic cancer over time. Furthermore, the lack of association between H2-antagonists and pancreatic cancer diminish the possibility of indication bias as an explanation for our findings.

Drawing causal relationships in the PPI versus cancer story is challenging [43], in particular because PPIs are by far the most commonly prescribed drug for most of its’ indications. In Sweden, long-term use of PPIs is almost 30 times more common than H2-receptor antagonists use when disregarding those using both drug classes during the study period [43]. Some of the indications may be unrecognized risk factors for pancreatic cancer, or actually be early symptoms of pancreatic cancer. This may explain the very high SIRs during the first year of follow-up, indicating reverse causality or protopathic bias, in particular among the youngest age group who may seem unlikely to be at risk for pancreatic cancer (potentially delaying diagnosis). Yet, all individuals required an estimated accumulated duration of exposure of at least 6 months. This should have eliminated those diagnosed shortly after initiating PPI treatment, yet an increased risk during that first year was not unlikely as also seen in our previous papers on gastric and esophageal cancer (with 7–9 times increased SIRs compared to the background population) [35]. Since pancreatic cancer is a relatively aggressive cancer (with 80–85% presenting with locally advanced or distant metastatic disease at time of diagnosis [45], it is however unlikely that early symptoms have been unrecognized longer than 1 year. In the early stage, most patients are asymptomatic [46]. Previous studies described abdominal pain in 25% of individuals up to 6 months prior to diagnosis [47], and only few symptoms occurring more than 6 months before diagnosis: back pain, shoulder pain, dysphagia, changes in bowel habits, and lethargy [46, 48].

This strongly reduces the risk of reverse causality in those analyses for the time periods after the first year. Importantly, the lack of association between H2-antagonists (with similar indications as PPI) and pancreatic cancer further strengthen the hypothesis that long-term PPI use may be carcinogenic. Finally, the increase in risk over time after this first year, both overall and for adenocarcinoma only, also supports our hypothesis that PPI may be an independent risk factor for pancreatic cancer.

As mentioned above, some epidemiological studies have been conducted to investigate the association between PPI use and pancreatic cancer, with 3 out of 7 studies showing strongly increased risks [27, 29, 30], and 4 without any statistical significant difference [28, 31–33]. Previous epidemiological studies also described increased risks of peri-ampullary cancer, cholangitis and cholecystitis [49–51]. Pre-clinical studies have shown that PPIs can create hypoacidity and consequently hypergastrinemia which may result in an overgrowth of pancreatic cells [33, 52]. PPIs have also been described as the drug-group with the largest effect on the lower gut microbiome based on population-based studies [53–56]. Yet, by reducing gastric acidity in the stomach, the bactericidal effect will also be reduced, leading to changes in the microbiome of the upper-gastrointestinal tract or bacterial overgrowth of potential harmful bacteria [56]. Regarding the results of the present study, and our previous studies on gastric and esophageal cancer showing similar age-dependent effects, a more thorough investigation of the effect of PPIs on the microbiome for different age-groups seems warranted. The clinical implications of this apparent increased relative risk of pancreatic cancer may be limited since the absolute, life-time risk of pancreatic cancer is low (approximately 1.5% [57]. Yet, since pancreatic cancer is usually diagnosed late, usually in individuals between 60–80 years [46], it may be important to be aware of this risk in particular in young long-term PPI users, because the symptoms are vague, and may suggest a gastro-esophageal origin. Unfortunately, the available data did not allow a more in depth analysis of duration/cumulative dosage among the youngest age-group because of the low absolute risk.

To conclude, this paper provides evidence for an independent association between long-term PPI use and the risk of pancreatic cancer, based on a large, population-based nationwide cohort study. Especially the strong increase among the youngest age-groups warrant attention, since this may indicate a stronger risk among young individuals and a higher risk of delayed diagnosis if PPIs are initiated and continued for early symptoms without more thorough examination.

## Abbreviations

CI: Confidence interval
H2RA: Histamine-2 receptor antagonist
PPI: Proton pump inhibitor
SIR: Standardized incidence ratio
